# The ameliorating effect of Rutin on hepatotoxicity and inflammation induced by the daily administration of vortioxetine in rats

**DOI:** 10.1186/s12906-024-04447-9

**Published:** 2024-04-05

**Authors:** Mai M. Anwar, Ibrahim M. Ibrahim Laila

**Affiliations:** 1 Department of Biochemistry, National Organization for Drug Control and Research (NODCAR)/Egyptian Drug Authority (EDA), Cairo, Egypt; 2 Department of Biotechnology &Molecular drug evaluation, National Organization for Drug Control and Research (NODCAR)/Egyptian Drug Authority (EDA), Cairo, Egypt

**Keywords:** SSRIs, Hepatotoxicity, Liver injury, DNA fragmentation, Apoptosis, Oxidative stress

## Abstract

**Background:**

Vortioxetine (VORTX) is a potent and selective type of selective serotonin reuptake inhibitor (SSRI) that is mainly prescribed for treating major depression along with mood disorders as the first drug of choice. Limited previous findings have indicated evidence of liver injury and hepatotoxicity associated with daily VORTX treatment. Rutin (RUT), which is known for its antioxidant properties, has demonstrated several beneficial health actions, including hepatoprotection. Therefore the current study aimed to evaluate and assess the ameliorative effect of RUT against the hepatotoxic actions of daily low and high-dose VORTX administration.

**Methods:**

The experimental design included six groups of rats, each divided equally. Control, rats exposed to RUT (25 mg/kg), rats exposed to VORTX (28 mg/kg), rats exposed to VORTX (28 mg/kg) + RUT (25 mg/kg), rats exposed to VORTX (80 mg/kg), and rats exposed to VORTX (80 mg/kg) + RUT (25 mg/kg). After 30 days from the daily exposure period, assessments were conducted for serum liver enzyme activities, hepatotoxicity biomarkers, liver antioxidant endogenous enzymes, DNA fragmentation, and histopathological studies of liver tissue.

**Results:**

Interestingly, the risk of liver damage and hepatotoxicity related to VORTX was attenuated by the daily co-administration of RUT. Significant improvements were observed among all detected liver functions, oxidative stress, and inflammatory biomarkers including aspartate aminotransferase (AST), alanine transaminase (ALT), lactate dehydrogenase (LDH), albumin, malondialdehyde (MDA), superoxide dismutase (SOD), glutathione (GSH), glutathione S-transferase (GST), total protein, acid phosphatase, N-Acetyl-/β-glucosaminidase (β*-NAG*), β-Galactosidase (β-Gal), alpha-fetoprotein (AFP), caspase 3, and cytochrom-C along with histopathological studies, compared to the control and sole RUT group.

**Conclusion:**

Thus, RUT can be considered a potential and effective complementary therapy in preventing hepatotoxicity and liver injury induced by the daily or prolonged administration of VORTX.

**Supplementary Information:**

The online version contains supplementary material available at 10.1186/s12906-024-04447-9.

## Background

Depression is a highly persistent, recurrent, and chronic disorder type. It is highly ranked as one of the leading causes of worldwide disability, affecting millions of people [1]. Depression symptoms are diverse and encompass a range of experiences such as low mood, loss of appetite, lack of interest migraine, cognitive impairment, and reduced energy. These directly impact the daily social life of individuals with depression and contribute significantly to the overall disease burden [1]. Additionally, depression results in direct alteration of the maintained balance between antioxidants and relative reactive oxygen species (ROS) by increasing oxidative stress levels. Antidepressant drugs are considered the most widely used drugs among the population. However, prolonged and excessive use of these drugs can lead to various risk factors and drawbacks, including liver injury, malnutrition-related chronic liver injury, inflammatory conditions, and HIV infection [2–6]. The selective serotonin reuptake inhibitor drug type (SSRI) is one of the most widely used antidepressant drugs which was first introduced in the early 80s for the alleviation of major depressive disorders, are associated with common and repetitive adverse effects, including hepatotoxicity and sexual dysfunction [7–9].

Drug-induced chronic liver injury is considered the fourth leading cause of liver damage among the population, and increasingly becoming a relevant matter of concern for physicians. Despite previously published data on antidepressant-induced liver injuries being relatively scarce, several patients treated with antidepressant drugs may develop hepatotoxicity and hepatitis on prolonged misuse [3, 10]. Liver damage is mainly unpredictable and idiosyncratic, and can also be influenced by factors such as drug dosage and duration of use. Depressed patients who are on SSRIs are more predisposed to liver injuries [9, 11]. Vortioxetine (VORTX) is a type of multimodal drug that modulates 5-hydroxytryptamine (5-HT) receptors and acts as an SSRI [11, 12]. It is highly indicated for the treatment of major depressive disorders. On the other hand, VORTX indirectly modulates glutamate and Gamma-aminobutyric acid (GABA) receptors by reducing both transmission [12]. Previous studies have indicated that SSRIs can lead to liver injuries, hepatitis, hepatotoxicity, and inflammation when used inappropriately or for extended periods [3, 9, 13, 14].

Flavonoids are a relatively large group type of polyphenolic compounds that play a crucial role in detoxifying free radicals and are highly abundant in vegetables, fruits, and medicinal plants. Rutin (RUT) is a famous type of glycosidic flavonoid and is more highly absorbed by humans than aglycones and can be found in tomato leaves, apples, onions, and tea [15–19]. The hepatoprotective, anti-inflammatory, and antioxidant effects of rutin are mainly attributed to its metabolite, quercetin. Upon daily oral administration of rutin, glycoside hydrolysis occurs, releasing quercetin metabolite [20]. The various pharmacological actions of rutin have been repeatedly stated among previously published preclinical and clinical studies. Rutin has previously shown therapeutic efficacy in various disease models such as rheumatoid arthritis, inflammatory bowel disease, inflammation, and metabolic syndrome, all of were attributed to its immunological and anti-inflammatory properties regulating pathways including nuclear factor kappa B (NF-κB), phosphoinositide 3-kinase (PI3K/Akt), mitogen-activated protein kinases (MAPK), Heme oxygenase-1 (HO-1), and Nuclear factor erythroid 2-related factor 2 (Nrf2) [20]. The administration of rutin also revoked the hepatotoxicity effects of the paclitaxel chemotherapeutic drug. It was shown that paclitaxel exerts different inflammatory actions by exaggerating the release of numerous inflammatory cytokines such as interleukin-17 A, interferon (INF), and tumor necrosis factor-alpha (TNF-α) [21]. Meanwhile [21], stated that rutin daily administration protected the liver from damageable effects by ameliorating elevated liver enzymes, and oxidative stress along with knocking off NF-κB and TNF-α receptors. It was also reported that rutin exerts a protective effect against DNA damage due to its wide antioxidant potential actions. Additionally, rutin administration boosted the antioxidative stress, anti-apoptotic, and anti-inflammatory defense mechanisms against doxorubicin toxicity in rats by suppressing TNF-α and regulating the Nrf2 transcription factor [22]. The daily base rutin administration was reported to protect diabetic patients from different symptoms along with attenuating cytotoxicity and oxidative stress in human erythrocytes [23, 24]. also reported that rutin revoked the nephrotoxicity induced by valproic acid administration in rats by suppressing the release of the signal transducer and activator of transcription 3, BAX, Janus Kinase 2, and caspase-3 levels along with increasing BCL2 expression. Rutin was also reputed to ameliorate intestinal toxicity and peptic ulcer by exerting antihistaminic actions, increasing the production of prostaglandin, and exhibiting antioxidant capacity in addition to scavenging oxidative stress [25]. It was also previously demonstrated that rutin exerts synergistic effects along with the daily administration of vitamin C in decreasing MDA, triglycerides, TNF-α, and C-reactive protein (CRP) in addition to exerting a protective effect against DNA damage among severe hemodialysis patients [26]. Therefore, the present study aims to investigate the main hepatoprotective actions of RUT against the hepatotoxic effects of VORTX administration in rats at different pharmacological doses in addition to highlighting its role in hindering and alleviating the drawbacks of VORTX-induced liver injury.

## Materials and methods

### Materials

Vortioxetine hydrobromide (VORTX-catalogue name: SML3388) was purchased and obtained from Sigma Chemical, Germany. Rutin hydrate (RUT) was also purchased and obtained from Sigma Chemical, Germany. All other used chemicals were freshly prepared and of high analytical grade.

### Animals and experimental design

The necessary required permission was obtained from the Ethical Committee of the National Organization of Drug Control and Research (NODCAR) approval number (NODCAR/II/8/2023) guided by the required 3Rs principles (Replacement, Reduction, and Refinement).The health and physical fitness condition of the experimental animals used in this study were highly monitored throughout the whole experimental design. Prior to the commencement of the study, all rats underwent a thorough health assessment by a licensed veterinarian to ensure they were free from any pre-existing undetected health conditions. Before the start of the experimental design, the thirty-six male healthy Albino rats of weight (150–170 gram), and age (9–10 weeks) were all weighed to calculate their initial weight and then were equally divided into six required groups as follows:

1. **Control Rats (G1)**: The included rats administrated saline solution orally for three weeks.
2. **Rats exposed to RUT (G2)**: The included rats administrated RUT at a dose of 25 mg/kg/daily/orally for three weeks with certain modifications [27].
3. **Rats exposed to VORTX low dose (G3)**: The included rats administrated VORTX at a dose of 28 mg/kg/daily/orally for three weeks with certain modifications [28].
4. **Rats exposed to VORTX low dose + RUT (G4)**: The included rats concurrently administrated VORTX at a dose of 28 mg/kg/daily/orally [28] and RUT at a dose of 25 mg/kg/daily/orally [27] for three weeks with certain modifications.
5. **Rats exposed to VORTX high dose (G5)**: The included rats administrated VORTX at a dose of 80 mg/kg/daily/orally for three weeks with certain modifications [29].
6. **Rats exposed to VORTX high dose + RUT (G6)**: The included rats concurrently administrated VORTX at a dose of 80 mg/kg/daily/orally [29] and RUT at a dose of 25 mg/kg/daily/orally [27] for three weeks with certain modifications.

Twenty-four hours after the last experimental design dosage administration, the final rats’ body weight for all the enclosed groups was directly weighted using automatic balance. Required blood samples were taken from rats’ retro-orbital veins. Obtained blood was centrifuged for 10 min at 4℃-3500 rpm. The serum was further stored at -20℃ for the biological assessment. Immediately after the rat’s decapitation under the effect of isoflurane anesthesia (2–3% in 100% oxygen) [30], the liver was isolated from each decapitated rat and directly washed with phosphate buffer saline. Whereas each liver was divided into two parts, the first part was for the preparation of the tissue homogenate according to the required biological assessment and manufacturer instructions, while the other part was fixed in 10% formalin and ethanol respectively for histopathological studies. Different isolated liver supernatant of the prepared liver tissue homogenate were stored for different assessments.

### Serum biological assessment

Alanine aminotransferase (ALT), Aspartate aminotransferase (AST), Albumin (Alb), and Total Protein (TP) kits were regularly detected in the obtained serum from all groups according to Spectrum-Diagnostics (Cairo, Egypt) manufacturer instructions. Lactate dehydrogenase was estimated using UV spectrophotometry via following the decrease in NADH at 340 nm according to Berger [31] and Kjeld [32].

### Detection of oxidative stress and antioxidants enzymes activities

Each isolated liver was directly homogenized in KCL buffer 1.15% (1:10) and then centrifuged at 1000xg (+ 4 C) for 15 min. MDA was detected in supernatants according to Placer et al. [33] and Mihara and Uchiyama [34] in correlation with the thiobarbituric acid reaction measured at 532 nm. Meanwhile, the homogenate for GSH analysis was centrifuged at 9,000 rpm xg and detected according to Sedlak and Lindsay [35] and Beutler et al. [36]. On the other hand, for the detection of SOD and GST, isolated liver parts were homogenized in KH_2_PO_4_ buffer + 1 mmol EDTA (pH 7.4) and then centrifuged at 12,000 xg for 30 min at 4 °C. The collected supernatant was used for the enzymatic and protein assessment. The Protein concentration was detected using standard Bovine serum albumin. SOD activity was detected according to Kakkar et al. [37], while GST phase II metabolizing enzyme was determined according to Habig et al. [38].

### Determination of lysosomal enzyme activities (LEAs)

Lysosomal Enzyme Activities (LEAs) including Acid phosphatase (ACP), β- galactosidase (β-GAL), and β-N-acetyl glucosaminidase (β-NAG) were estimated and determined by spectrophotometry according to Van Hoof and Hers [39].

### Detection of inflammatory and apoptotic factors

Capsase-3 and Cytochrome-C (Cyt-C) were detected and estimated in prepared liver tissue homogenate according to manufacturer instructions (MyBioSource). Meanwhile, Alpha-fetoprotein (AFP) was detected and estimated in serum according to manufacturer instructions (MyBioSource).

### Estimation and detection of DNA damages

Detection of hepatic DNA fragmentation was estimated according to Wu et al. [40] and Trerè et al. [41]. DNA Ladder presence was detected according to Wlodek et al. [42]. Meanwhile, DNA extraction was conducted according to Aljanabi and Martinez [43]. Electrophoresis gel was prepared using agarose 2% containing (200 µg/ml) ethidium bromide 0.1%. Loading buffer (bromophenol blue 0.25%, xylene 0.25%, and glycerol 30%) was highly mixed with different DNA samples and was directly loaded into different wells (20 µl DNA per lane) with a special standard ladder marker. Whereas, the prepared gel was electrophoresed at a 50 mA/1.5 h estimated current using an electrophoresis machine. The DNA was visualized and photographed using UV light illumination. We wish to note that, due to technical constraints during the imaging process, full-length gel images were not obtainable for inclusion in this manuscript. However, it is essential to emphasize that the absence of these images did not compromise the integrity or validity of our results. The gel blot images presented in this study accurately reflect the findings obtained from our experiments. All relevant images, including additional gel blot images, have been provided as supplementary material accompanying this manuscript. Readers are encouraged to refer to the supplementary file for a comprehensive overview of the experimental data and procedures described in this study.

### Histological analysis of isolated liver tissue

Isolated liver tissues were first washed and then directly fixed in formalin solution 10% for 2 days. Fixed tissues were then dehydrated in a prepared graded series of ethanol. Different samples were stained with hematoxylin and eosin (H&E) followed by being examined under light microscopy. Detection of pathological changes was mainly based on the presence of hepatocellular necrosis, disarrangement of hepatic cells, and the degree of hepatic nuclear asymmetry.

### Statistical analysis

Observed data are expressed in the form of mean ± standard error. Statistical analysis was conducted using a one-way analysis of variance, followed by Dunnett’s test to assess the degree of statistical. *P* < 0.05 level was considered statistically significant.

## Results

### Effect of Rutin (RUT) and Vortioxetine (VORTX) on the final body and liver estimated weights

Rats exposed to VORTX low (G3) and high doses (G5) showed a significant reduction in estimated body weight and liver size when compared to control (G1) and rats exposed to RUT (G2) (*P* < 0.05). Meanwhile, body and liver organ weights were slightly restored following the exposure to RUT in both groups of rats exposed to VORTX high dose (G6) and VORTX low dose (G4) when compared to control (G1) and rats exposed to RUT (G2) (*P* < 0.05), indicating the ameliorating effect of RUT against VORTX-induced hepatotoxicity as shown in (Table 1).

**Table 1:** The alleviative role of RUT against VORTX toxicity on organ and body weight

Groups	Body weight (g) Mean ± SE		Liver weight (g)
	Initial	Final	
**Control Rats (G1)**	158.333 **±** 3.158^$^	268.833 **±** 7.586^@^	1.921 **±** 0.020^@^
**Rats exposed to RUT (G2)**	158.167 **±** 2.257^$^	264.833 **±** 5.594^@^	1.935 **±** 0.020^@^
**Rats exposed to VORTX low dose (G3)**	160 **±** 4.049^$^	200.833 **±** 3.360^$^	1.656 **±** 0.054^#^
**Rats exposed to VORTX low dose + RUT (G4)**	160.5 **±** 5.981^$^	240.666 **±** 3.480^#^	1.755 **±** 0.045^@^
**Rats exposed to VORTX high dose (G5)**	165.5 **±** 6.026^$^	187.166 + 1.301^$^	1.186 **±** 0.052^#^
**Rats exposed to VORTX high dose + RUT (G6)**	164.666 **±** 5.713^$^	199 **±** 3.651^$^	1.41 **±** 0.051^#^

Values are expressed as mean ± SE of 6 rats per group. Values with the same superscript symbols are non-significant at (P>0.05) RUT = Rutin, Vortioxetine = VORTX

### Alanine aminotransferase (ALT), Aspartate aminotransferase (AST), Albumin (Alb), Total Protein (TP), and Lactate dehydrogenase serum levels following the exposure to VORTX different doses and RUT treatment

**Fig. 1:**
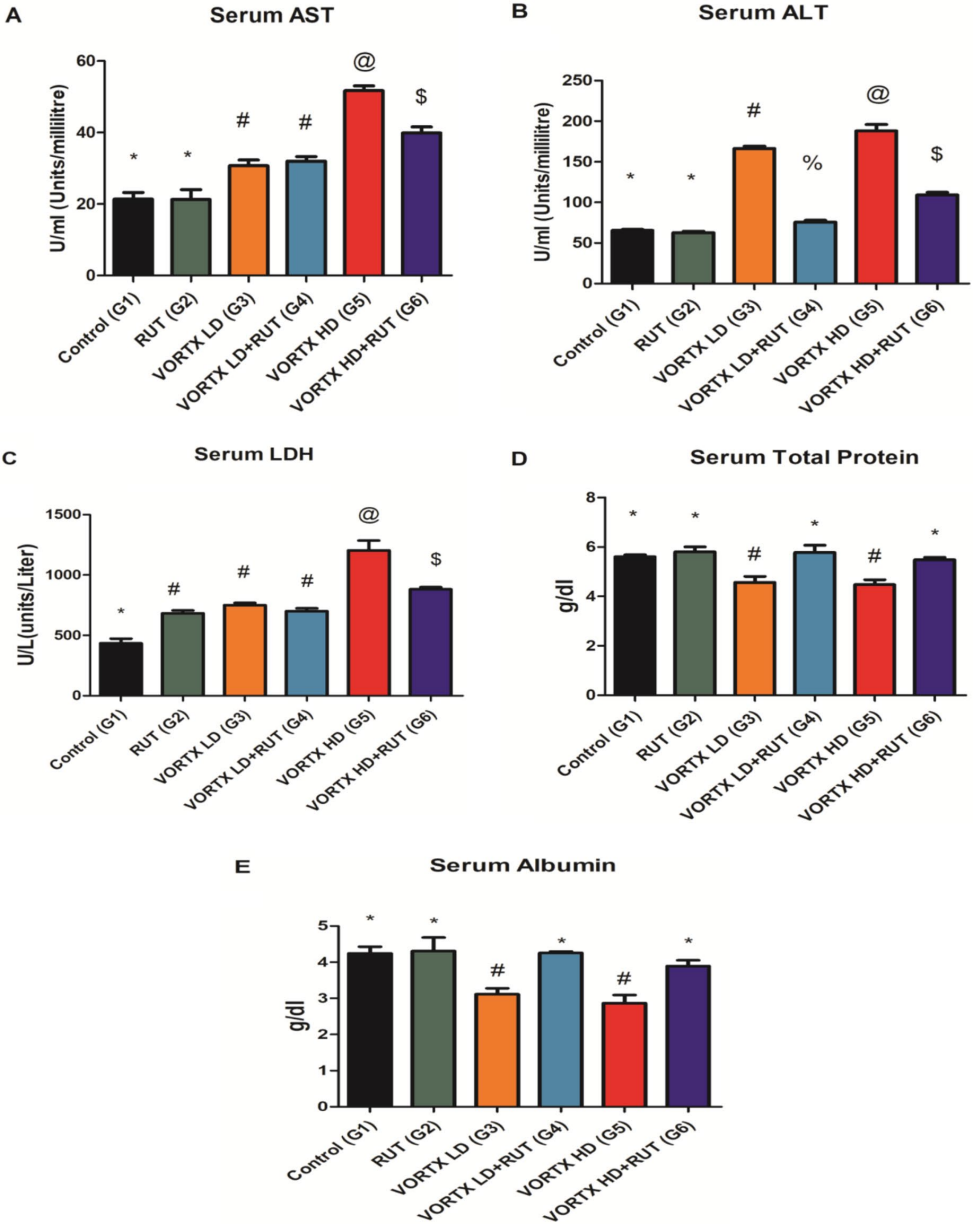
The alleviative role of RUT against VORTX toxicity on (**A**) AST, (**B**) ALT, (**C**) LDH, (**D**) Total protein, and (**E**) Albumin levels among exposed rats. Values are expressed as mean ± SE of 6 rats per group. Values with the same symbols are non-significant at (*P* > 0.05) RUT = Rutin, Vortioxetine = VORTX, Low dose = LD, High dose = HD

Serum AST, ALT, and LDH levels were estimated and given as shown in (Fig. 1). The observed data demonstrated that the exposure to VORTX low (G3) and high doses (G5) resulted in a significant increase in the serum levels of AST, ALT, and LDH when compared to control (G1) and rats exposed to RUT (G2) (*P* < 0.05). This indicates the degree and severity of hepatocytes damage, especially among group (G5). On the other hand, it was highly observed that serum albumin and total protein levels were significantly decreased following the exposure to both doses of VORTX (G3 & G5) with more relevant effects in the group of rats exposed to high VORTX dose (G5). However, it was demonstrated that administration of RUT in both groups of rats exposed to low (G4) and high VORTX doses (G6) protected the liver tissues from severe cellular damage by restoring adequate levels of AST, ALT, LDH, albumin, and total protein when compared to control (G1) and rats exposed to RUT (G2) (*P* < 0.05) as shown in (Fig. 1).

### Status of oxidative stress and antioxidant markers following the exposure to VORTX different doses and RUT treatment

**Fig. 2:**
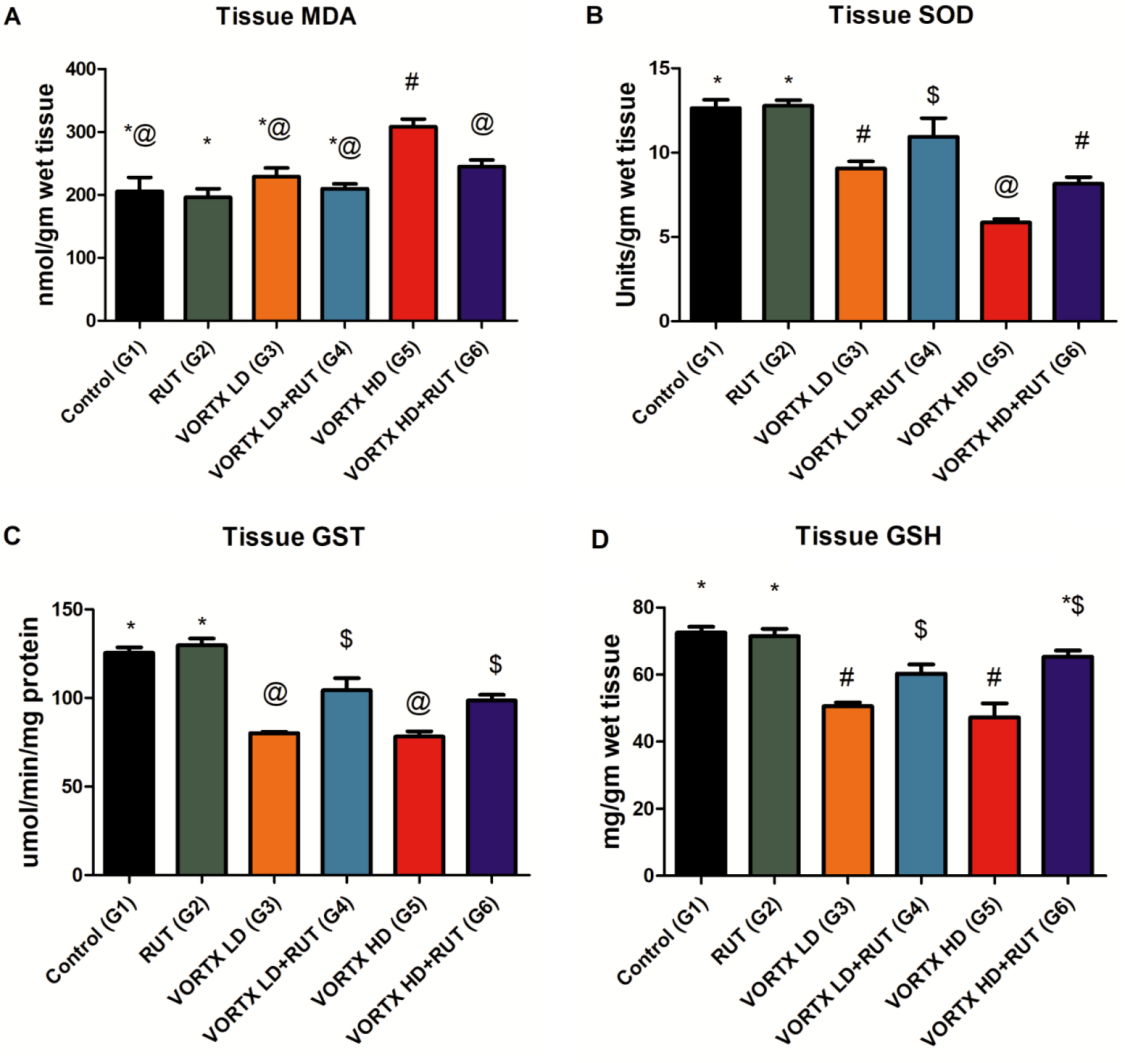
The alleviative role of RUT against VORTX toxicity on (**A**) MDA, (**B**) SOD, (**C**) GST, and (**D**) GSH tissue levels among exposed rats. Values are expressed as mean ± SE of 6 rats per group. Values with the same symbols are non-significant at (*P* > 0.05) RUT = Rutin, Vortioxetine = VORTX, Low dose = LD, High dose = HD

In the current study, MDA, SOD, GST, and GSH levels were detected for the estimation of oxidative stress in liver tissues following the exposure to low and high doses of VORTX (G3&G5, respectively) as represented in (Fig. 2). According to our observed data, the administration of VORTX in low (G3) and high doses (G5) increased the MDA level in liver tissues and suppressed SOD, GST, and GSH activities in liver tissues when compared to control (G1) and rats exposed to RUT (G2) (*P* < 0.05). Moreover, it was observed that RUT administration among both groups (G4 & G6) alleviated the induced oxidative stress and free radicles levels as a drawback of daily VORTX administration by decreasing MDA levels associated with increasing SOD, GST, and GSH liver tissue levels when compared to control (G1) and rats exposed to RUT (G2) (*P* < 0.05) as shown in (Fig. 2). This emphasizes the fact that the daily VORTX administration has a significant damaging effect on the liver tissues by inducing oxidative damage to hepatocytes leading to severe hepatotoxicity.

### Status of inflammatory and apoptotic markers following the exposure to VORTX different doses and RUT treatment

**Fig. 3:**
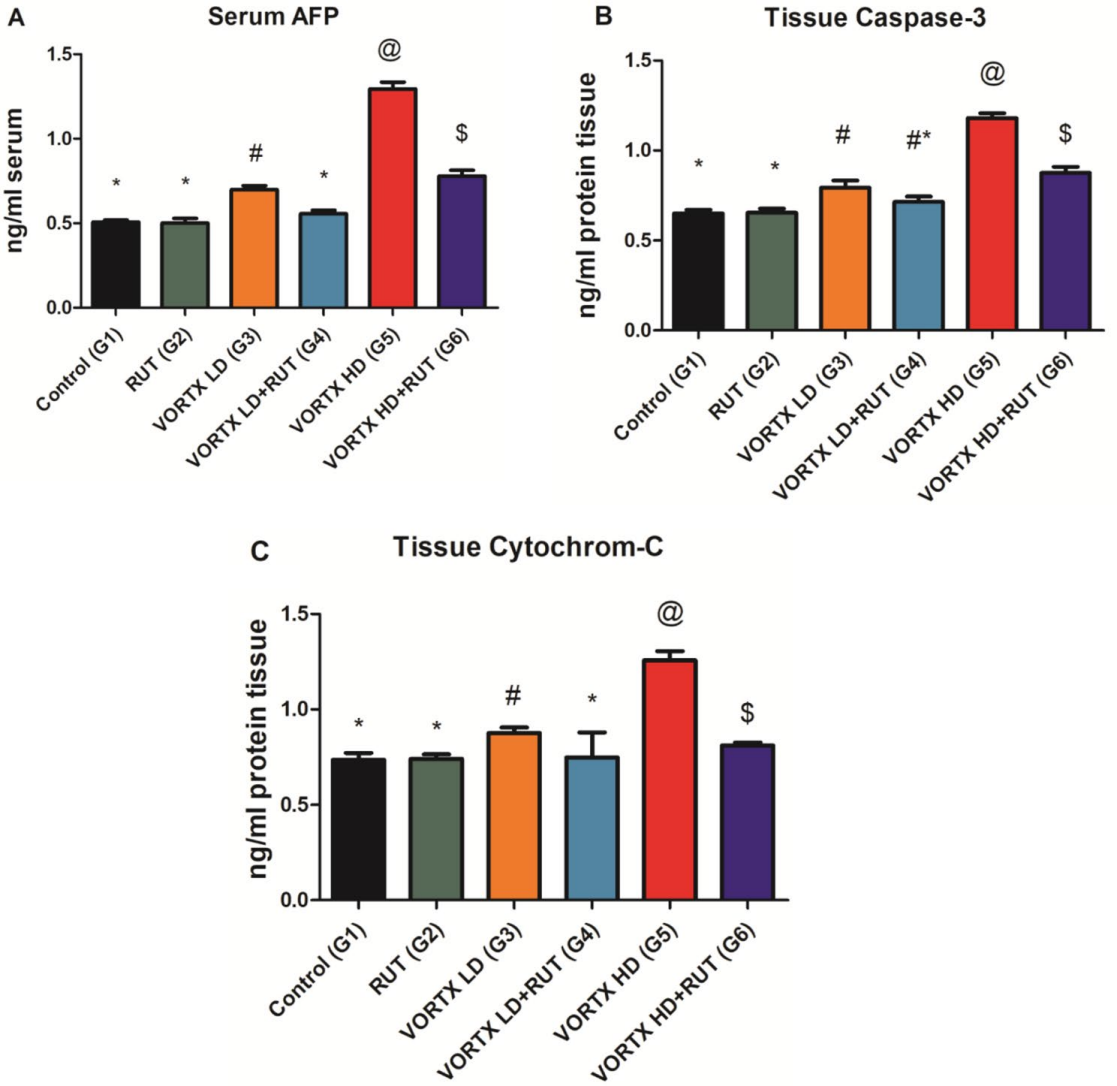
The alleviative role of RUT against VORTX toxicity on (**A**) AFP, (**B**) Caspase-3, and (**C**) Cytochrome-C levels among exposed rats. Values are expressed as mean ± SE of 6 rats per group. Values with the same symbols are non-significant at (*P* > 0.05) RUT = Rutin, Vortioxetine = VORTX, Low dose = LD, High dose = HD

To evaluate the effects of VORTX misuse on the inflammatory markers, AFP was detected in serum while Cytochrome-C and Caspase 3 were detected in liver tissues. According to the obtained results represented in (Fig. 3), it was determined that the administration of VORTX in low (G3) and high doses (G5) resulted in elevated AFP, caspase 3, and cytochrome C levels among all the exposed group when compared to control (G1) and rats exposed to RUT (G2) (*P* < 0.05). These observed results highlight the degree of inflammation and apoptosis induced as a drawback of VORTX daily administration among exposed groups, especially in high-dose VORTX group (G5). Whereas, the administration of RUT among rats exposed to VORTX in low and high doses (G4, G6 respectively) resulted in major improvement among all the inflammatory estimated parameters when compared to control (G1) and rats exposed to RUT (G2) (*P* < 0.05) as shown in (Fig. 3).

### Status of lysosomal enzymes activities (LAEs) following the exposure to VORTX different doses and RUT treatment

**Fig. 4:**
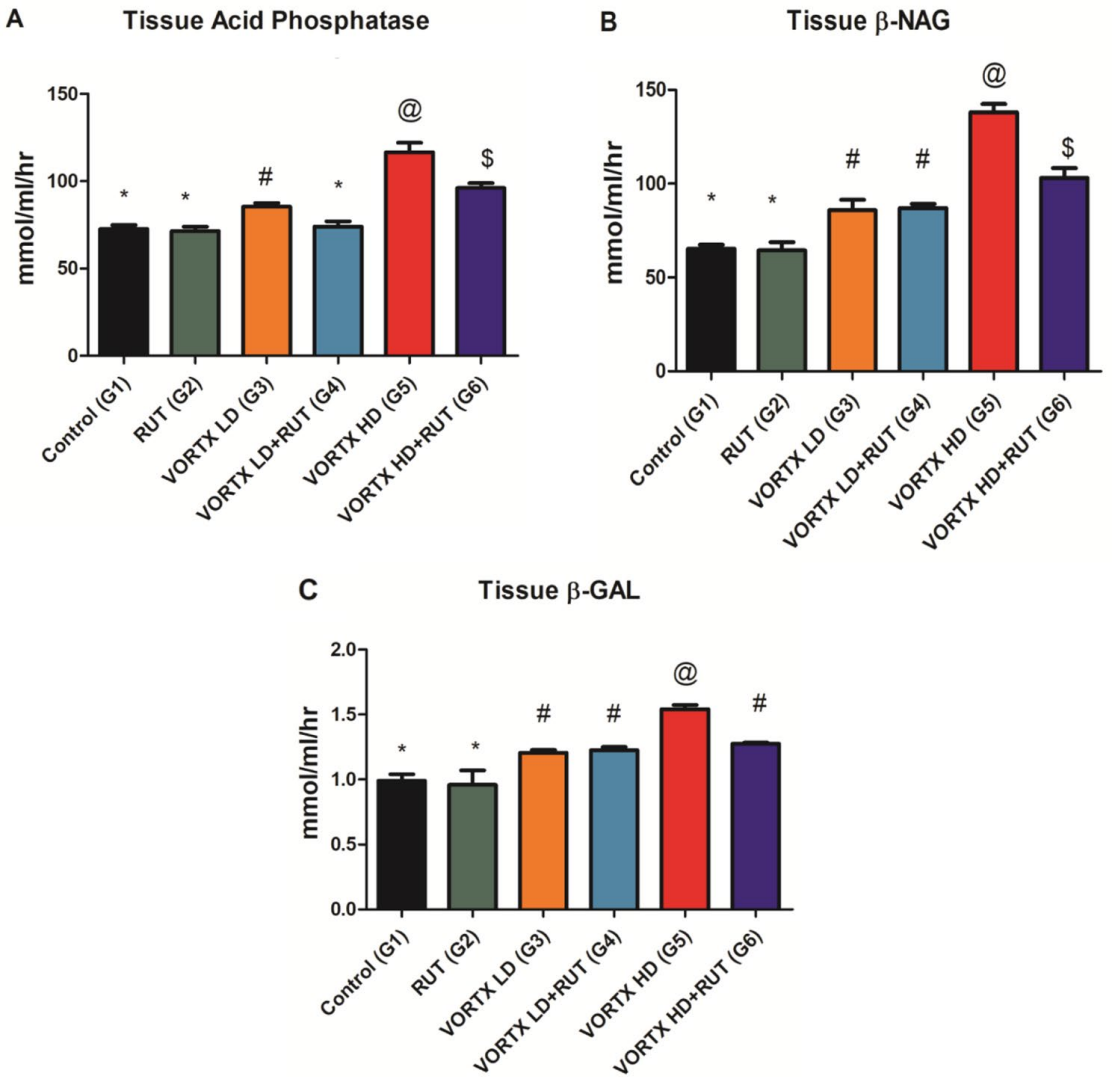
The alleviative role of RUT against VORTX toxicity on (**A**) Acid phosphatase (**B**) B-NAG, and (**C**) B-GAL, levels among exposed rats. Values are expressed as mean ± SE of 6 rats per group. Values with the same symbols are non-significant at (*P* > 0.05) RUT = Rutin, Vortioxetine = VORTX, Low dose = LD, High dose = HD

The effect of VORTX administration on the liver lysosomal enzyme activities represented in the form of (Acid phosphatase, B-NAG, and B-GAL) was demonstrated in (Fig. 4). It was concluded that VORTX administration, especially among the group of rats exposed to high VORTX dose (G5) significantly increased the release level and activities of these three enzymes when compared to control (G1) and rats exposed to RUT (G2) (*P* < 0.05). The Administration of RUT significantly ameliorated the effect of VORTX on both low and high doses when compared to control (G1) and rats exposed to RUT (G2) (*P* < 0.05) as shown in (Fig. 4).

### DNA fragmentation analysis and purity following the exposure to VORTX different doses and RUT treatment

**Fig. 5:**
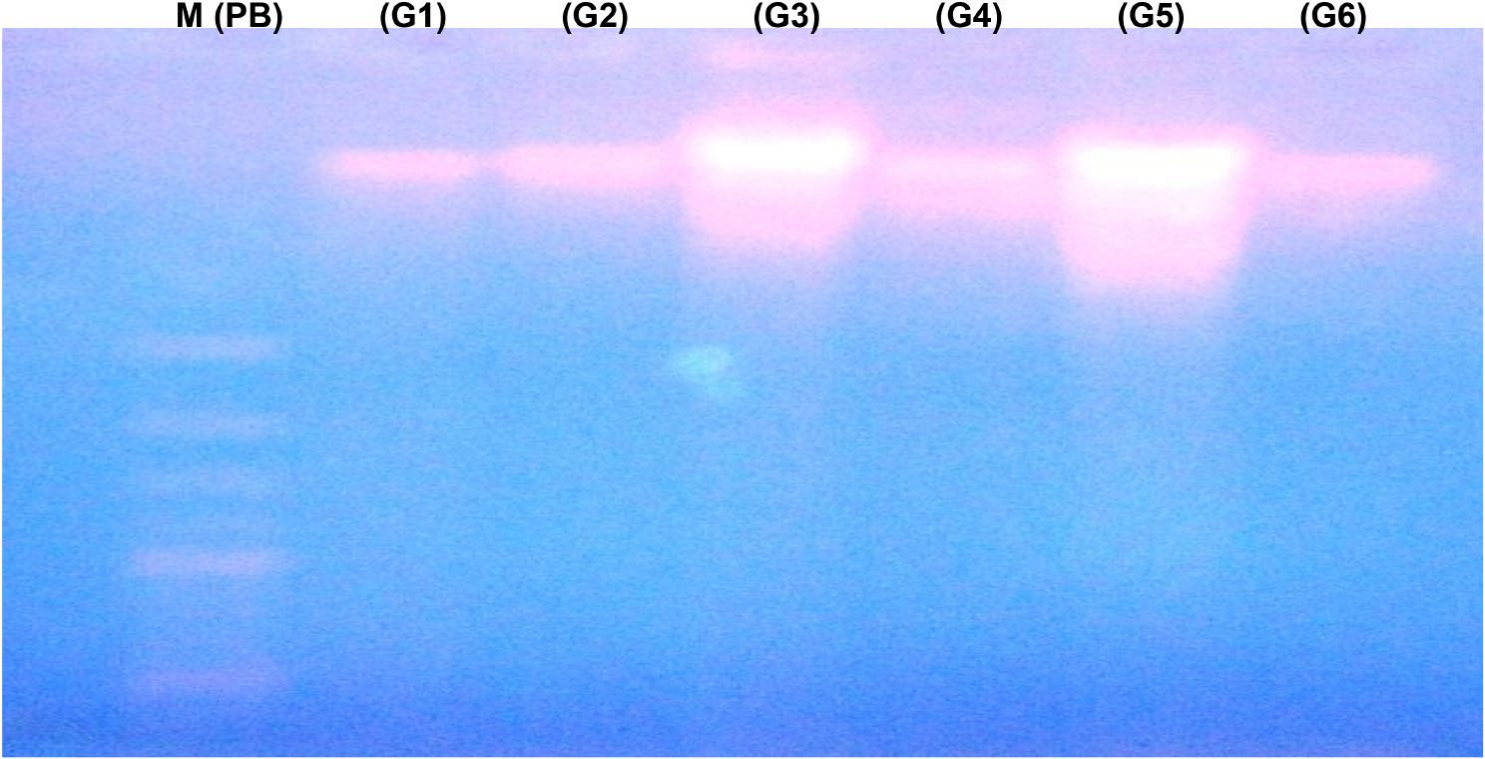
The alleviative role of RUT against VORTX toxicity on DNA fragmentation of liver tissues among all exposed rats. Agarose gel electrophoretic isolated DNA pattern of liver tissues among all exposed rats. Lane 1 from Left: (Marker 2.5kbp), Lane 2: (control G1), Lane 3: (Rats exposed to RUT G2), Lane 4: (Rats exposed to VORTX low dose G3), Lane 4: (Rats exposed to VORTX low dose + RUT G4), Lane 5: (Rats exposed to VORTX high dose G5), and Lane 6: (Rats exposed to VORTX high dose + RUT G6)

Table 2 illustrates the degree of DNA purity and the concentration of total dsDNA among all the exposed six groups of rats to estimate the degree of VORTX-induced hepatotoxicity and the alleviative role of RUT against the hepatic damage induced by daily VORTX administration at different pharmacological doses. The normal estimated DNA purity should be between (1.8 to 2.0) at the absorbance of (260 nm) indicating that nucleic acid (NA) is free from any contamination. As demonstrated in our observed data, DNA purity and total dsDNA concentration were detected to be decreased following the exposure to VORTX low dose (G3) and (G5) with a more significant decrease among the high dose exposed group (G5). On the other hand, DNA purity and total dsDNA concentration were increased among both RUT-treated groups + VORTX low/High doses (G4 & G6, respectively) when compared to control (G1) and rats exposed to RUT (G2) (*P* < 0.05). Moreover, the degree of DNA fragmentation was detected and estimated by gel electrophoresis technique represented in the form of DNA ladder constituting series of fragments (180–200 bp) as shown in (Fig. 5). An observed increase in the degree of DNA fragmentation was observed following the exposure to VORTX low (G3) and high doses (G5) with a more significant increase in the degree of fragmentation among the group of rats exposed to high VORTX dose (G6) when compared to control (G1) and rats exposed to RUT (G2) (*P* < 0.05). Treatment with RUT resulted in a detected significant improvement in the degree of DNA fragmentation among (G4 & G6) when compared to control (G1) and rats exposed to RUT (G2) (*P* < 0.05) as demonstrated in Table 2.

**Table 2:** The alleviative role of RUT against VORTX toxicity on DNA purity and total ds DNA concentration in rats’ liver tissues

Groups	Mean ± SE	
	Purity A260/A280	Total dsDNA Concentration (mg/ml)
**Control Rats (G1)**	1.9216 **±** 0.0205^@^	4.5566 **±** 0.0923^@^
**Rats exposed to RUT (G2)**	1.9350 **±** 0.0202^@^	4.5066 **±** 0.0899^@^
**Rats exposed to VORTX low dose (G3)**	1.6566 **±** 0.0543^#^	4.025 **±** 0.0808^$^
**Rats exposed to VORTX low dose + RUT (G4)**	1.755 **±** 0.0452^#^	4.3216 **±** 0.1014^@$^
**Rats exposed to VORTX high dose (G5)**	1.1866 **±** 0.0520^$^	2.1666 **±** 0.1473^%^
**Rats exposed to VORTX high dose + RUT (G6)**	1.41 **±** 0.0516^%^	3.3216 **±** 0.1711^&^

Values are expressed as mean ± SE of 6 rats per group. Values with the same superscript symbols are non-significant at (P>0.05) RUT = Rutin, Vortioxetine = VORTX

### Histopathological structure

**Fig. 6:**
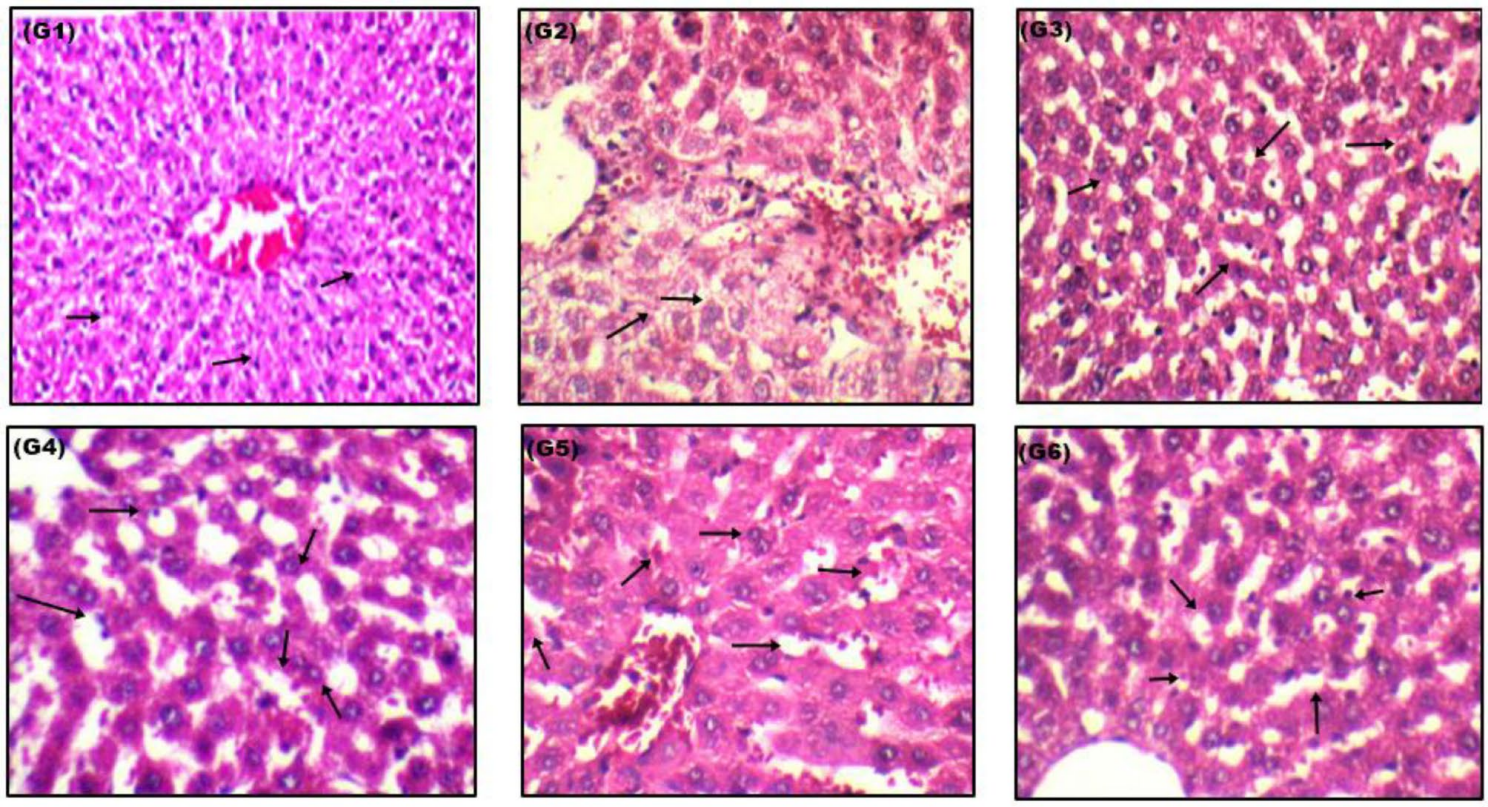
Hematoxylin and eosin (H&E)-stained liver sections among all exposed groups (control G1): showed normal hepatocyte structures (400X), (Rats exposed to RUT G2): showing normal intact liver appearance (640X), (Rats exposed to VORTX low dose G3): showing inflammatory hepatocyte cells infiltration (640X), (Rats exposed to VORTX low dose + RUT G4): showing slight hepatocyte vacuolation and inflammation (640X), (Rats exposed to VORTX high dose G5): showing relevant congested, thickened and scattered portal/central veins (640X), and (Rats exposed to VORTX high dose + RUT G6): showing very slight hepatocyte vacuolation and mild degree of congested sinusoid (640X)

The light microscopic examinations of the isolated liver sections of the control (G1) (Fig. 6) showed normal hepatocyte structure with intact nucleus and cisternae of rough endoplasmic reticulum (RER). The isolated liver sections of the RUT exposed group (G2) revealed normal hepatocytes with intact and healthy Kupffer cells. However, the isolated liver sections of rats exposed to VORTX low dose (G3) showed hepatocytes with congested blood vessels along with extravasated RBCs. Swollen vacuolated hepatocytes were also detected around the central veins associated with aggregated fatty cells. Moreover, congested dilated sinusoids with brown pigments were detected. The isolated liver section of the group of rats exposed to low VORTX dose + RUT (G4) showed mild inflammatory cell aggregates detected in some portal areas together with prominent Kupffer cells. Almost normal hepatocyte structures was detected with a limited number of vacuolated hepatocyte structures in focal areas. On the other hand, relevant congested, thickened, and scattered portal/central veins with proliferated bile ducts were detected among the group of rats exposed to VORX high dose (G5). Additionally, severe inflammatory aggregated cells, dilated congested sinusoids, and micro-vacuolated hepatocytes were also detected. Moreover, the isolated liver sections of rats exposed to high VORTX dose + RUT (G6) showed a relevant picture of regenerated normal hepatocytes. A mild degree of a congested sinusoid in limited areas was detected along with extravasated RBCs structure and very slight intact Kupffer cells. An improvement in the degree of aggregated inflammatory cells among different areas was also observed.

## Discussion

Drug-induced hepatotoxicity and liver injury represent adverse reactions to the type of administrated drug and its metabolites, potentially leading to irreversible and prominent inadequacy in liver functions. These conditions encompass a wide range of manifestations and consequences, that range from asymptomatic abnormalities (silent stage) to symptomatic (severe symptoms and disabilities) [44]. Liver injury resulting from the drawbacks of drug abuse or misuse can be divided into two types: intrinsic and idiosyncratic. Intrinsic hepatotoxicity is dose-dependent, characterized by a short latency period, and follows a predictable disease time [45]. On the other hand, idiosyncratic hepatotoxicity is not dose-dependent, but exhibits variable manifestations, and has an unpredictable disease course [46]. The lack of specific biological markers often hinders early diagnosis of liver injury and hepatotoxicity. Clinical symptoms are diverse and may differ from one patient to another including loss of appetite, tiredness, fever, vomiting, jaundice, and muscle pain. In most cases, immediate treatment involves discontinuing the offending drug and providing the necessary required medical support [47].

The liver serves as the primary organ responsible for the metabolism of antidepressant drugs. Thus, it is important to find out how prolonged administration or overdoses of these administrated drugs, along with their metabolites, may impact this vital organ. Hepatotoxicity may directly result in severe inflammation, fibrosis, steatohepatitis, hepatic steatosis, cirrhosis, and necrosis [18, 19, 48]. Even at maintained therapeutic doses, prolonged or misuse of antidepressant drugs may result in serious hepatotoxicity [47, 49, 50]. It was previously highlighted in one of the clinical studies that the administration of antidepressants and psychotropic drugs was responsible for 7.6% of the induced liver injuries among 185 subjects [51, 52]. Therefore, it is important to establish an outline and strategy for prescribing antidepressant drugs to any patient, taking into consideration their hepatotoxicity and other associated risk factors, including diabetes and drug abuse [47, 50]. The metabolism of antidepressant drugs and SSRIs, including VORTX, occurs predominantly in the liver, via the cytochrome P450-dependent monooxygenase which serves as the main drug-metabolizing enzyme for a range of drugs such as steroids, xenobiotics, and vitamins. The lipophilic character of most antidepressant drugs facilitates their easy transfer to the cell membrane and is primarily metabolized in the liver [53].

The prolonged or high-dose administration of VORTX can lead to significant liver damage via different mechanisms, including direct hepatic toxicity, inflammation, apoptosis, oxidative stress, and DNA fragmentation, ultimately resulting in impaired hepatic function and structural alterations [54]. Oxidative stress and inflammation are the two main driving factors of hepatotoxicity and liver injury. As demonstrated in our results, the administration of VORTX in low and high doses results in an exaggerated increase in oxidative stress, and apoptotic factors in addition to a relevant decrease in antioxidants level. Consistent with our results, it was reported that the administration of VORTX and SSRIs induces oxidative stress and inflammation in the liver tissues by promoting apoptotic factors, ROS production, DNA damage, and lipid peroxidation, associated with suppressed oxidative stress release [54–57]. One of the main sources of elevated ROS in the liver is CYP450 and mitochondria in hepatocytes [58]. Additionally, Kupffer cells, immune cells, and neutrophils contribute to ROS production. ROS directly introduce carbonyl group compounds into several amino acid side chains, affecting DNA and protein structures and functions. Furthermore, Oxidative stress may directly oxidize polyunsaturated fatty acids type in the cascade of lipid peroxidation. ROS also results in DNA mutation, and a decrease in DNA purity and expression [59, 60]. This justifies that the misuse or prolonged administration of VORTX may directly lead to a direct reinforcement of DNA, which is likely an adverse consequence of triggered oxidative stress and the release of exaggerated inflammatory factors in affected liver tissues.

Meanwhile, the observed deficit in GSH release serves as a relevant indicator of the liver pro-oxidant state and the degree of hepatotoxicity [61]. Accordingly, the observed decrease in the level and the activity of GSH, GST, and SOD associated with an increase in MDA activity level along with apoptotic factors and lysosomal enzymes, can be attributed to the administration of VORTX in both low and high doses. In agreement with our results [62–66], demonstrated that the administration of SSRIs resulted in a severe increase in oxidative stress and apoptotic factor levels in the liver tissue including caspase-3, Bax, AFP, and cytochrome-c, mediating severe damaging effects in hepatocytes. It was demonstrated in the current study the administration of VORTX resulted in a significant increase in liver function serum level biomarkers including AST and ALT along with LDH. On the other hand, a relevant decrease in the serum levels of albumin and total protein was also observed following the administration of VORTX especially in high doses. The elevation of these biomarkers indicates the degree of liver injuries and the state of hepatocyte damage. In accordance with our results, the administration of SSRIs resulted in severe liver injuries indicated by relevant elevation of liver function biomarkers in addition to relevant histopathological studies [62, 63, 65–70].

Interestingly, VORTX administration triggers substantial inflammatory responses as a drawback of elevated oxidative stress levels and ROS. The marked elevated levels in the inflammatory responses are reflected in the expression levels of caspase-3, cytochrome-c, and AFP. The increase in oxidative stress and ROS production directly affects membrane permeability and mitochondrial functions, resulting in server hepatocyte damage and initiating apoptotic cell death via different apoptotic pathways. These triggered apoptotic and inflammatory responses include toll-like receptors (TLR) and nuclear factor kappa B (NF-κB) [65, 66, 71, 72]. Consequently, the observed decrease in the DNA purity and total dsDNA concentration may be a consequence of increased ROS and oxidative stress in depression patients and those receiving antidepressants [65–67, 71, 56, 73, 74]. The detected histopathological alterations following the administration of VORTX, especially at high doses, including relevant inflammatory cell infiltrations, hepatocytes vacuolation, highly dilated sinusoids, and other several manifestations can be attributed to apoptosis, inflammation, and exaggerated oxidative stress.

Rutin, a flavonol compound which is abundantly found in different plants was demonstrated to have several pharmacological activities, including anti-inflammatory, antioxidant, vasoprotective, and cytoprotective [17, 75]. Flavonoids are majorly converted to different metabolites by the action of intestinal microflora and specific liver enzymes. Upon administration of rutin, it is actively converted to quercetin and other types of metabolites [27]. The hepatoprotective efficiency of RUT is evident through the notable reduction in liver enzyme activities, oxidative stress, apoptotic factors, DNA damage, and lysosomal enzymes induced by previous exposure to VORTX, especially at high doses [27, 76, 77]. Whereas, nearly normal levels of liver enzyme biomarkers, oxidative stress, antioxidants, inflammatory factors, total DNA, and histopathological studies were relevantly restored in RUT-treated groups pre-exposed to VORTX in both low and high doses. In line with our results, previous studies have demonstrated the hepatoprotective efficiency of RUT due to its various pharmacological properties, especially antioxidant, antiapoptotic, and anti-inflammatory [27, 73, 76, 77]. Since the administration of RUT restored the antioxidant and anti-inflammatory levels, relevant improvements in total dsDNA concentration and DNA purity levels were also detected in our observed levels. Meanwhile, the histopathological studies revealed that VORTX administration resulted in severe hepatic tissue damage supported by severe swelling, inflammation, and hepatocytes. These histopathological and pathological changes were restored following RUT administration indicating the relevant protective efficacy of RUT on liver morphology.

## Conclusion

Since inadequate responses to prolonged or misused administration of antidepressants have been repeatedly seen due to various and unexpected side effects of drugs, new therapeutic approaches are urgently required. Our results reveal that the daily administration of Vortioxetine (VORTX) at both low and high doses can lead to varying degrees of liver injury and hepatotoxicity as a drawback of inflammation, elevated oxidative stress, apoptosis, and DNA damage. Importantly, our study demonstrates that Rutin (RUT) exhibits promising effects as a hepatoprotective agent in case administered as part of a daily routine. Rutin shows potential in mitigating VORTX-induced hepatotoxicity by restoring normal liver functions, reducing levels of ROS, alleviating inflammation, and suppressing apoptotic factors, all while bolstering the body’s defenses against oxidative stress. The current study sheds new insights on the drawbacks associated with the daily administration of VORTX, particularly in relation to its hepatotoxic effects. Moreover, it emphasizes the positive impact of daily RUT flavone glycoside administration in counteracting the induced hepatic damage. Thus, these findings may influence clinical decision-making, especially in cases where VORTX is considered the drug of choice. Further clinical research should be effectively designed to assess the effectiveness of Rutin supplementation in preventing VORTX-induced hepatotoxicity.

In addition to the implications for clinical practice highlighted above, future research endeavors should aim to deepen our understanding of the hepatoprotective properties of Rutin and evaluate its potential utility in preventing antidepressant-induced hepatotoxicity. Longitudinal studies are warranted to assess the long-term safety and efficacy of Rutin supplementation in mitigating VORTX-induced liver injury. Furthermore, comparative studies evaluating the safety profiles of various antidepressants and the impact of adjunctive therapies on hepatic function are essential for guiding treatment decisions and optimizing patient outcomes.

### Electronic supplementary material

Below is the link to the electronic supplementary material.

Supplementary Material 1

## Data Availability

The obtained data analyzed during the current study are available from the corresponding author on reasonable request.

## Abbreviations

VORTX: Vortioxetine
RUT: Rutin
SSRIs: selective serotonin reuptake inhibitors
MD: major depression
(MDDs): major depressive disorders
LEAs: Lysosomal Enzyme Activities
RER: rough endoplasmic reticulum
TLR: toll-like receptors
NF-κB: nuclear factor kappa B

## References

[1] Liu S, Li C, Shi Z, Wang X, Zhou Y, Liu S (2017). Caregiver burden and prevalence of depression, anxiety and sleep disturbances in Alzheimer’s disease caregivers in China. J Clin Nurs.

[2] Chen M, Suzuki A, Borlak J, Andrade RJ, Lucena MI (2015). Drug-induced liver injury: interactions between drug properties and host factors. J Hepatol.

[3] Leise MD, Poterucha JJ, Talwalkar JA. Drug-induced liver injury. Mayo Clinic proceedings. 2014;89(1):95–106. 10.1016/j.mayocp.2013.09.016 24388027

[4] Murray CJL, Lopez AD (2013). Measuring the global burden of Disease. N Engl J Med.

[5] Elsebaie S, Abdel-Fattah EM, Bakr AN, Koh NA, Attalah KM, Aweas A-HA (2023). Principles of Nutritional Management in Patients with Liver Dysfunction&mdash;A Narrative Review. Livers.

[6] Shin S, Jun DW, Saeed WK, Koh DH (2021). A narrative review of malnutrition in chronic liver disease. Ann Transl Med.

[7] Anderson HD, Pace WD, Libby AM, West DR, Valuck RJ (2012). Rates of 5 common antidepressant side effects among New Adult and adolescent cases of Depression: a retrospective US claims Study. Clin Ther.

[8] Rush AJ, Trivedi MH, Wisniewski SR, Nierenberg AA, Stewart JW, Warden D (2006). Acute and longer-term outcomes in depressed outpatients requiring one or several treatment steps: a STAR*D report. Am J Psychiatry.

[9] Spigset O, Hägg S, Bate A (2003). Hepatic injury and pancreatitis during treatment with serotonin reuptake inhibitors: data from the World Health Organization (WHO) database of adverse drug reactions. Int Clin Psychopharmacol.

[10] Danan G, Teschke R (2019). Roussel Uclaf Causality Assessment Method for Drug-Induced Liver Injury: Present and Future. Front Pharmacol.

[11] Hong X, Zhang L, Zha J (2022). Toxicity of waterborne vortioxetine, a new antidepressant, in non-target aquatic organisms: from wonder to concern drugs?. Environ Pollut.

[12] Stahl SM (2015). Modes and nodes explain the mechanism of action of vortioxetine, a multimodal agent (MMA): enhancing serotonin release by combining serotonin (5HT) transporter inhibition with actions at 5HT receptors (5HT1A, 5HT1B, 5HT1D, 5HT7 receptors). CNS Spectr.

[13] Mullish BH, Kabir MS, Thursz MR, Dhar A (2014). Review article: depression and the use of antidepressants in patients with chronic liver disease or liver transplantation. Aliment Pharmacol Ther.

[14] Edinoff AN, Akuly HA, Hanna TA, Ochoa CO, Patti SJ, Ghaffar YA (2021). Selective serotonin reuptake inhibitors and adverse effects: a narrative review. Neurol Int.

[15] Domitrović R, Jakovac H, Vasiljev Marchesi V, Vladimir-Knežević S, Cvijanović O, Tadić Z (2012). Differential hepatoprotective mechanisms of rutin and quercetin in CCl(4)-intoxicated BALB/cN mice. Acta Pharmacol Sin.

[16] Hafez MM, Al-Harbi NO, Al-Hoshani AR, Al-Hosaini KA, Al Shrari SD, Al Rejaie SS (2015). Hepato-protective effect of rutin via IL-6/STAT3 pathway in CCl4-induced hepatotoxicity in rats. Biol Res.

[17] Rahmani S, Naraki K, Roohbakhsh A, Hayes AW, Karimi G (2023). The protective effects of rutin on the liver, kidneys, and heart by counteracting organ toxicity caused by synthetic and natural compounds. Food Sci Nutr.

[18] Anwar MM, Laila IMI (2022). Mitigative effect of caffeine against diclofenac-induced hepato-renal damage and chromosomal aberrations in male albino rats. BMC Complement Med Ther.

[19] Anwar MM, Laila IMI. Protective and restorative potency of diosmin natural flavonoid compound against tramadol-induced testicular damage and infertility in male rats. Nat Prod Res. 2022:1–5. 10.1080/14786419.2022.2090937 35730634

[20] González R, Ballester I, López-Posadas R, Suárez MD, Zarzuelo A, Martínez-Augustin O (2011). Effects of flavonoids and other polyphenols on inflammation. Crit Rev Food Sci Nutr.

[21] Ali YA, Soliman HA, Abdel-Gabbar M, Ahmed NA, Attia KAA, Shalaby FM (2023). Rutin and Hesperidin revoke the Hepatotoxicity Induced by Paclitaxel in male Wistar rats < i > via their Antioxidant, anti-inflammatory, and antiapoptotic activities. Evidence-Based Complement Altern Med.

[22] Ahmed OM, Elkomy MH, Fahim HI, Ashour MB, Naguib IA, Alghamdi BS (2022). Rutin and Quercetin Counter Doxorubicin-Induced Liver toxicity in Wistar rats < i > via their Modulatory effects on inflammation, oxidative stress, apoptosis, and Nrf2. Oxidative Med Cell Longev.

[23] Salam S, Arif A, Sharma M, Mahmood R (2023). Protective effect of rutin against thiram-induced cytotoxicity and oxidative damage in human erythrocytes. Pestic Biochem Physiol.

[24] Kandemir FM, Ileriturk M, Gur C (2022). Rutin protects rat liver and kidney from sodium valproate-induce damage by attenuating oxidative stress, ER stress, inflammation, apoptosis and autophagy. Mol Biol Rep.

[25] Gautam R, Singh M, Gautam S, Rawat JK, Saraf SA, Kaithwas G (2016). Rutin attenuates intestinal toxicity induced by Methotrexate linked with anti-oxidative and anti-inflammatory effects. BMC Complement Altern Med.

[26] Omar S, El Borolossy RM, Elsaid T, Sabri NA. Evaluation of the combination effect of rutin and vitamin C supplementation on the oxidative stress and inflammation in hemodialysis patients. Front Pharmacol. 2022;13. 10.3389/fphar.2022.961590 PMC9493033 36160426

[27] Pan P-H, Lin S-Y, Wang Y-Y, Chen W-Y, Chuang Y-H, Wu C-C (2014). Protective effects of rutin on liver injury induced by biliary obstruction in rats. Free Radic Biol Med.

[28] Sharp AM, Lertphinyowong S, Yee SS, Paredes D, Gelfond J, Johnson-Pais TL et al. Vortioxetine reverses medial prefrontal cortex-mediated cognitive deficits in male rats induced by castration as a model of androgen deprivation therapy for prostate cancer. 2019;236(11):3183–95. 10.1007/s00213-019-05274-4 PMC6832770 31139875

[29] Qin M, Qiao H-q, Yuan Y-j, Shao Q (2018). A quantitative LC-MS/MS method for simultaneous determination of deuvortioxetine, vortioxetine and their carboxylic acid metabolite in rat plasma, and its application to a toxicokinetic study. Anal Methods.

[30] Bhatia A, Saikia PP, Dkhar B, Pyngrope H (2022). Anesthesia protocol for ear surgery in Wistar rats (animal research). Anim Models Experimental Med.

[31] Berger R. Methods of enzymatic analysis (3rd edition). Volume III Enzyme 1: Oxidoreductases, Transferases., Beach W. Florida/Basel: Verlag Chemie 1983., 605 S., 18 Abb., 43 Table 224 DM (wenn alle Bände), 258 DM (Einzelband). Acta Biotechnologica. 1984;4(4):346-.

[32] Kjeld M (1972). An automated colorimetric method for the estimation of lactate dehydrogenase activity in serum. Scand J Clin Lab Investig.

[33] Placer ZA, Cushman LL, Johnson BC (1966). Estimation of product of lipid peroxidation (malonyl dialdehyde) in biochemical systems. Anal Biochem.

[34] Mihara M, Uchiyama M (1978). Determination of malonaldehyde precursor in tissues by thiobarbituric acid test. Anal Biochem.

[35] Sedlak J, Lindsay RH (1968). Estimation of total, protein-bound, and nonprotein sulfhydryl groups in tissue with Ellman’s reagent. Anal Biochem.

[36] Beutler E, Duron O, Kelly BM (1963). Improved method for the determination of blood glutathione. J Lab Clin Med.

[37] Kakkar P, Das B, Viswanathan PN (1984). A modified spectrophotometric assay of superoxide dismutase. Indian J Biochem Biophys.

[38] Habig WH, Pabst MJ, Jakoby WB (1974). Glutathione S-transferases. The first enzymatic step in mercapturic acid formation. J Biol Chem.

[39] van Hoof F, Hers HG (1968). The Abnormalities of Lysosomal Enzymes in Mucopolysaccharidoses. Eur J Biochem.

[40] Wu B, Ootani A, Iwakiri R, Sakata Y, Fujise T, Amemori S (2006). T cell deficiency leads to liver carcinogenesis in azoxymethane-treated rats. Experimental biology and medicine. (Maywood NJ).

[41] Trerè D, Zilbering A, Dittus D, Kim P, Ginsberg PC, Daskal I (1996). AgNOR quantity in needle biopsy specimens of prostatic adenocarcinomas: correlation with proliferation state, Gleason score, clinical stage, and DNA content. Clin Mol Pathol.

[42] Wlodek D, Banáth J, Olive PL (1991). Comparison between pulsed-field and constant-field gel electrophoresis for measurement of DNA double-strand breaks in irradiated Chinese Hamster ovary cells. Int J Radiat Biol.

[43] Aljanabi SM, Martinez I (1997). Universal and rapid salt-extraction of high quality genomic DNA for PCR-based techniques. Nucleic Acids Res.

[44] Chalasani N, Fontana RJ, Bonkovsky HL, Watkins PB, Davern T, Serrano J (2008). Causes, clinical features, and outcomes from a prospective study of Drug-Induced Liver Injury in the United States. Gastroenterology.

[45] Roth RA, Ganey PE (2010). Intrinsic versus idiosyncratic drug-induced hepatotoxicity–two villains or one?. J Pharmacol Exp Ther.

[46] Jee A, Sernoskie SC, Uetrecht J (2021). Idiosyncratic Drug-Induced Liver Injury: mechanistic and clinical challenges. Int J Mol Sci.

[47] Navarro VJ, Senior JR (2006). Drug-related hepatotoxicity. N Engl J Med.

[48] Anwar MM, Mabrouk AA. Hepatic and cardiac implications of increased toxic amyloid-beta serum level in lipopolysaccharide-induced neuroinflammation in rats: new insights into alleviating therapeutic interventions. Inflammopharmacology. 2023. 10.1007/s10787-023-01202-3 PMC10229490 37017850

[49] Aithal G, Watkins P, Andrade R, Larrey D, Molokhia M, Takikawa H (2011). Case definition and phenotype standardization in Drug-Induced Liver Injury. Clin Pharmacol Ther.

[50] Mu W, Xu G, Wei Z, Wang Z, Qin Q, Lin L (2022). The role of NLRP3 inflammasome in psychotropic drug-induced hepatotoxicity. Cell Death Discovery.

[51] Licata A, Minissale MG, Calvaruso V, Craxì A (2017). A focus on epidemiology of drug-induced liver injury: analysis of a prospective cohort. Eur Rev Med Pharmacol Sci.

[52] Dumortier G, Cabaret W, Stamatiadis L, Saba G, Benadhira R, Rocamora JF (2002). [Hepatic tolerance of atypical antipsychotic drugs]. L’Encephale.

[53] Perlis RH (2007). Cytochrome P450 genotyping and antidepressants. BMJ (Clinical Res ed).

[54] Ye H, Nelson LJ, Gómez Del Moral M, Martínez-Naves E, Cubero FJ (2018). Dissecting the molecular pathophysiology of drug-induced liver injury. World J Gastroenterol.

[55] Battal D, Yalin S, Eker ED, Aktas A, Sahin NO, Cebo M (2014). Possible role of selective serotonin reuptake inhibitor sertraline on oxidative stress responses. Eur Rev Med Pharmacol Sci.

[56] Ștefan M-G, Kiss B, Gutleb AC, Loghin F (2020). Redox metabolism modulation as a mechanism in SSRI toxicity and pharmacological effects. Arch Toxicol.

[57] Boshtam S, Shokrzadeh M, Ghassemi-Barghi N (2023). Fluoxetine induces oxidative stress-dependent DNA damage in human hepatoma cells. AIMS Med Sci.

[58] Cesaratto L, Vascotto C, Calligaris S, Tell G (2004). The importance of redox state in liver damage. Ann Hepatol.

[59] Solek P, Mytych J, Tabecka-Lonczynska A, Sowa-Kucma M, Koziorowski M. Toxic effect of antidepressants on male reproductive system cells: evaluation of possible fertility reduction mechanism. J Physiol Pharmacol. 2021;72(3). 10.26402/jpp.2021.3.06 34810289

[60] BERK A, YILMAZ İ (2018). The Side effects of antidepressants and the Importance of Medicinal Plants in the treatment of Depression. Int J Med Sci Health Res.

[61] Han D, Hanawa N, Saberi B, Kaplowitz N (2006). Mechanisms of Liver Injury. III. Role of glutathione redox status in liver injury. Am J Physiology-Gastrointestinal Liver Physiol.

[62] Elgebaly HA, Mosa NM, Allach M, El-massry KF, El-Ghorab AH, Al Hroob AM (2018). Olive oil and leaf extract prevent fluoxetine-induced hepatotoxicity by attenuating oxidative stress, inflammation and apoptosis. Biomed Pharmacother.

[63] Schoretsanitis G, Carlini SV, John M, Kane JM, Deligiannidis KM (2021). Antenatal antidepressant prescription Associated with reduced fetal femur length but not estimated fetal weight: a retrospective Ultrasonographic Study. J Clin Psychopharmacol.

[64] Levkovitz Y, Gil-Ad I, Zeldich E, Dayag M, Weizman A (2005). Differential induction of apoptosis by antidepressants in glioma and neuroblastoma cell lines. J Mol Neurosci.

[65] Nastić K, Pecikoza U, Labudović-Borović M, Kotur-Stevuljević J, Micov A, Jovanović A (2023). The antidepressant drugs vortioxetine and duloxetine differentially and sex-dependently affect animal well-being, cognitive performance, cardiac redox status and histology in a model of osteoarthritis. Biomed Pharmacother.

[66] Zhan Y, Wang A, Yu Y, Chen J, Xu X, Nie J et al. Inhibitory mechanism of vortioxetine on CYP450 enzymes in human and rat liver microsomes. Front Pharmacol. 2023;14. 10.3389/fphar.2023.1199548 PMC10544575 37790811

[67] Voican CS, Corruble E, Naveau S, Perlemuter G (2014). Antidepressant-Induced Liver Injury: a review for clinicians. Am J Psychiatry.

[68] González-Muñoz M, Monserrat Villatoro J, Marín-Serrano E, Stewart S, Bardón Rivera B, Marín J (2020). A case report of a drug-induced liver injury (DILI) caused by multiple antidepressants with causality established by the updated Roussel Uclaf causality assessment method (RUCAM) and in vitro testing. Clin Case Rep.

[69] Chen M, Borlak J, Tong W (2013). High lipophilicity and high daily dose of oral medications are associated with significant risk for drug-induced liver injury. Hepatology (Baltimore MD).

[70] Telles-Correia D, Barbosa A, Cortez-Pinto H, Campos C, Rocha NB, Machado S (2017). Psychotropic drugs and liver disease: a critical review of pharmacokinetics and liver toxicity. World J Gastrointest Pharmacol Ther.

[71] Czarny P, Wigner P, Galecki P, Sliwinski T (2018). The interplay between inflammation, oxidative stress, DNA damage, DNA repair and mitochondrial dysfunction in depression. Prog Neuropsychopharmacol Biol Psychiatry.

[72] Todorović Vukotić N, Đorđević J, Pejić S, Đorđević N, Pajović SB (2021). Antidepressants- and antipsychotics-induced hepatotoxicity. Arch Toxicol.

[73] Gur C, Kandemir FM. Molecular and biochemical investigation of the protective effects of rutin against liver and kidney toxicity caused by malathion administration in a rat model. 2023;38(3):555–65. 10.1002/tox.23700 36346126

[74] Khan RA, Khan MR, Sahreen S (2012). CCl4-induced hepatotoxicity: protective effect of rutin on p53, CYP2E1 and the antioxidative status in rat. BMC Complement Altern Med.

[75] Ganeshpurkar A, Saluja AK (2017). The pharmacological potential of Rutin. Saudi Pharm J.

[76] Nafees S, Rashid S, Ali N, Hasan SK, Sultana S (2015). Rutin ameliorates cyclophosphamide induced oxidative stress and inflammation in Wistar rats: role of NFκB/MAPK pathway. Chemico-Biol Interact.

[77] Küçükler S, Kandemir FM, Özdemir S, Çomaklı S, Caglayan C (2021). Protective effects of rutin against deltamethrin-induced hepatotoxicity and nephrotoxicity in rats via regulation of oxidative stress, inflammation, and apoptosis. Environ Sci Pollut Res.

