# Supplementary material for: The ameliorating effect of Rutin on hepatotoxicity and inflammation induced by the daily administration of vortioxetine in rats
Source: BMC Complement Med Ther. 2024 Apr 5;24:153. doi: 10.1186/s12906-024-04447-9 (PMC10996088; doi:10.1186/s12906-024-04447-9)
Supplement: Supplementary file 1 — Supplementary Material 1 [file 12906_2024_4447_MOESM1_ESM.docx]

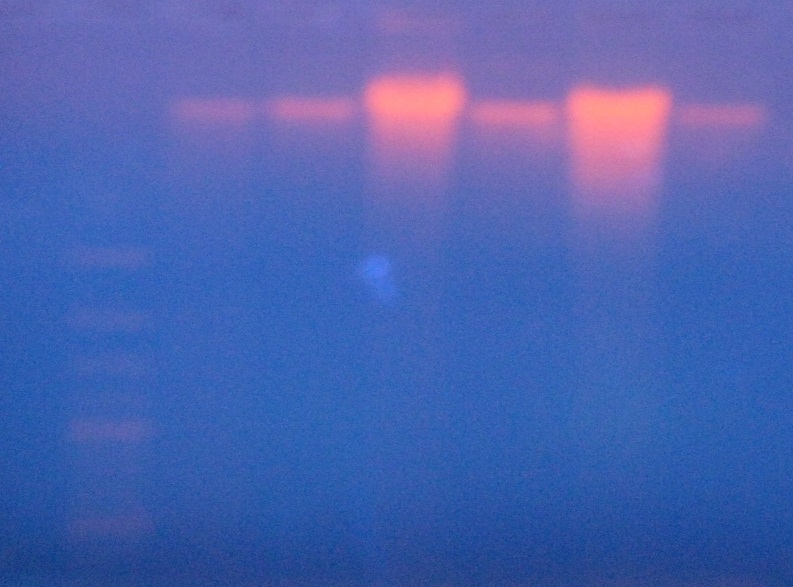


**M (PB) (G1) (G2) (G3) (G4) (G5) (G6)**

**M (PB) (G1) (G2) (G3) (G4) (G5) (G6)**


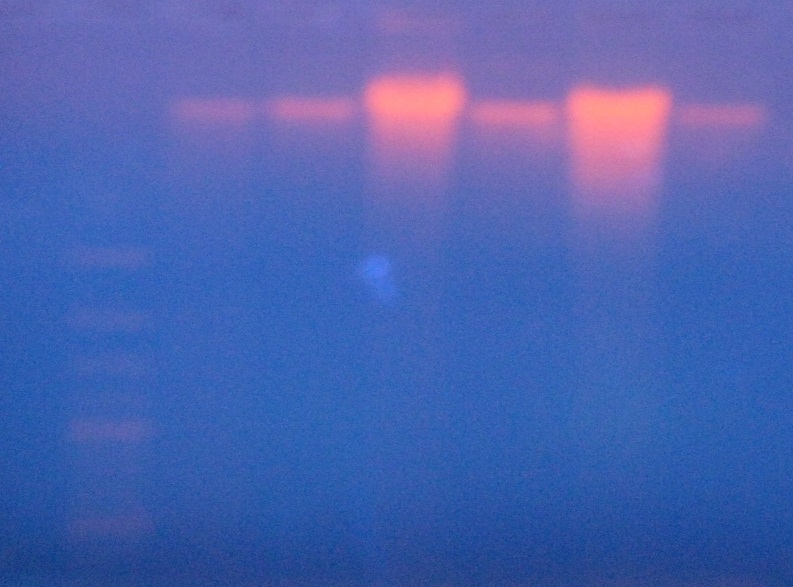


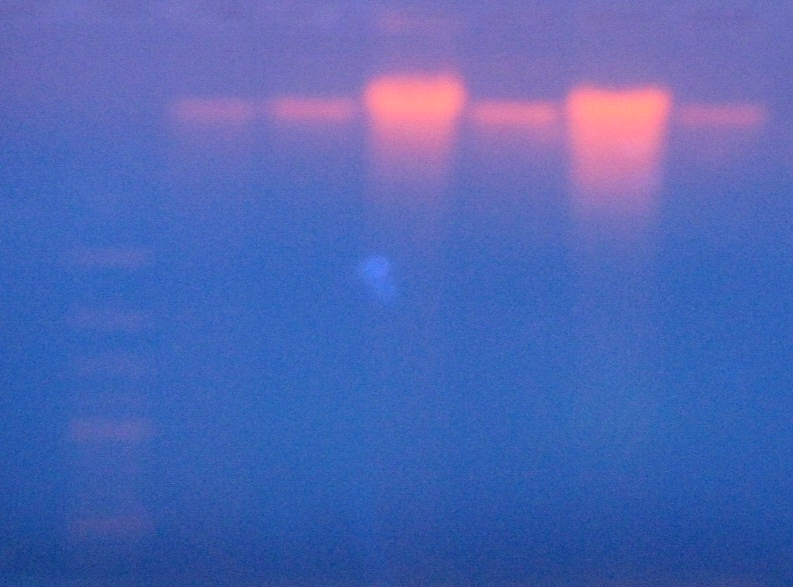


**M (PB) (G1) (G2) (G3) (G4) (G5) (G6)**

Figure S1: Presents all the available gel blot images for the Genomic DNA of liver tissue on agarose gel electrophoresis as a supplementary material. Figure represents the alleviative role of RUT against VORTX toxicity on DNA fragmentation of liver tissues among all exposed rats. Agarose gel electrophoretic isolated DNA pattern of liver tissues among all exposed rats. Lane 1 from Left: (Marker 2.5kbp), Lane 2: (control G1), Lane 3: (Rats exposed to RUT G2), Lane 4: (Rats exposed to VORTX low dose G3), Lane 4: (Rats exposed to VORTX low dose + RUT G4), Lane 5: (Rats exposed to VORTX high dose G5), and Lane 6: (Rats exposed to VORTX high dose + RUT G6). Please refer to the main text and Methods section for detailed descriptions of the experimental procedures and analyses.
